# Sexual-size dimorphism modulates the trade-off between exploiting food and wind resources in a large avian scavenger

**DOI:** 10.1038/s41598-017-11855-0

**Published:** 2017-09-13

**Authors:** Pablo A. E. Alarcón, Juan M. Morales, José A. Donázar, José A. Sánchez-Zapata, Fernando Hiraldo, Sergio A. Lambertucci

**Affiliations:** 1 Grupo de Investigaciones en Biología de la Conservación, INIBIOMA (Universidad Nacional del Comahue-CONICET), Quintral 1250 (R8400FRF), Bariloche, Argentina; 2 Grupo de Ecología Cuantitativa, INIBIOMA (Universidad Nacional del Comahue-CONICET), Quintral 1250 (R8400FRF), Bariloche, Argentina; 3 The Peregrine Fund. 668 West Flying Hawk Lane, Boise, ID 83709 USA; 4 Department of Conservation Biology, Estación Biológica de Doñana, CSIC, E-41092, Sevilla, Spain; 5 Department of Applied Biology, University Miguel Hernández, E-03202 Alicante, Spain

## Abstract

Animals are expected to synchronize activity routines with the temporal patterns at which resources appear in nature. Accordingly, species that depend on resources showing temporally mismatched patterns should be expected to schedule routines that balance the chances of exploiting each of them. Large avian scavengers depend on carcasses which are more likely available early in the morning, but they also depend on wind resources (i.e. uplifts) to subside flight which are stronger in afternoon hours. To understand how these birds deal with this potential trade-off, we studied the daily routines of GPS-tagged individuals of the world’s largest terrestrial soaring scavenger, the Andean condor (*Vultur gryphus*). Andean condors vary largely in weight and show a huge sexual dimorphism that allowed us to evaluate the effect of sex and body size on their daily routines. We found that condors use an intermediate solution strategy between the best times to exploit carcasses and uplifts, with this strategy changing over the year. Bigger males scheduled earlier routines that aligned more closely with uplift availability compared to smaller females, resulting in a partial temporal segregation between sexes. Condors’ routines reflect a sexual-size dependent trade-off that may underpin ecological and sociobiological traits of the studied population.

## Introduction

Performance trade-offs are thought to impose strong constraints on the adaptive evolution of animal behavior^1^. Broadly speaking, performance trade-offs occur when a beneficial change in one trait is necessarily linked to a detrimental change in some other trait^2, 3^. As a consequence, animal behavior cannot be optimized across the board as excellence in one behavioral trait will come at the cost of performance in another. This is how trade-offs often give rise to compromise behavioral phenotypes; i.e. individuals performing sub-optimally due to counteracting demands^4–7^.

Daily behavioral routines of animals are shaped by performance trade-offs. This is because, in order to make important decisions (e.g. when to feed), individuals need to evaluate costs and benefits in a multidimensional space in whose axes the variables of interest often show mismatched temporal patterns^8^. For instance, to search for food, animals choose times of the day that maximize the energy intake but taking into account potential penalties derived from encounters with predators, competitors and unfavorable weather conditions^9–11^. Because of this, when the environment drives an animal’s decisions towards opposite extremes, natural selection should then act on the individual to get the best balance between them^1, 12^.

Individual attributes can lead animals to find different trade-off solutions that ultimately shape daily routines of individuals. For instance, sexual differences may imply that males and females have different nutritional requirements, roles in offspring rearing and competitive abilities^13–15^ that result in a temporal niche partitioning between sexes^16, 17^. In the case of size-dimorphic soaring birds, males and females often show differences in flight performances that may have functional significance and lead sexes to exploit scenarios with different weather conditions^18, 19^. Similarly, trade-off solutions can be adjusted to the seasonal changes in physiology, weather and food availability leading daily routines to vary throughout the year^20–22^.

Large avian scavengers are expected to deal with an important trade-off when scheduling their daily routines. On the one hand, they depend on a sparsely distributed food resource (i.e. animal carcasses) that forces them to forage early in the morning in order to ensure time to find food and reduce the risk of starvation^20, 22–24^. In fact, it is early in the morning when the chances of finding a profitable carcass are highest because ungulate mortality commonly peaks during night time due to thermal stress or predation by nocturnal carnivores^25^. On the other hand, large avian scavengers strongly depend on wind resources in the form of uplifts as their body size makes powered flight energetically costly. Thus, they are expected to match up activities that require long-distance movements to particular weather conditions^12, 26, 27^. In terrestrial environments the morning hours typically do not present wind speeds and temperatures high enough to create profitable uplift conditions^28^ and, therefore, there are good reasons to think that these birds face a mismatch between the optimal times to exploit food and wind resources.

The Andean condor (*Vultur gryphus*) is an excellent model to explore the existence of this type of trade-offs as it is an obligate scavenger, the world’s largest terrestrial soaring bird and a sexually size-dimorphic species in which males are larger than females. In this study, we first hypothesize that the daily routines of GPS-tagged condors inhabiting the Andean Patagonia are shaped by a trade-off between the temporally mismatched needs of obtaining food and subsiding flight with wind resources. On the basis of this trade-off, the birds should decide among: i) foraging as early as possible in the morning to maximize the probability of finding food (‘food-prioritization strategy’), ii) foraging in mid-afternoon to minimize energy expenditure in locomotion by exploiting the most profitable wind resources (‘wind-prioritization strategy’) or iii) to reach a solution that is intermediate by foraging between the best time to exploit food and wind resources (‘intermediate solution strategy’) (Supplementary Fig. S1). As a result, the decisions under the first two strategies would be made at the expenses of relinquishing the optimal times of the day to exploit one of the resources, whereas the third one would imply to exploit both of them at the highest possible level given the constraints imposed by the trade-off.

**Figure 1:**
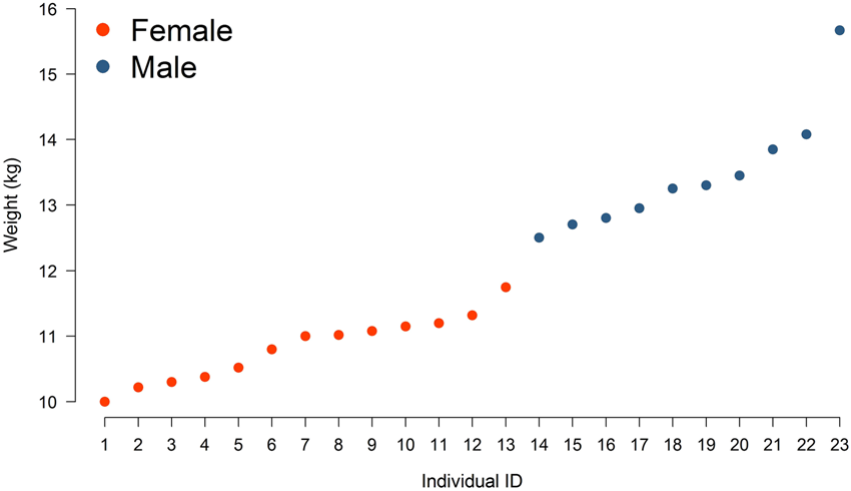
Relationship between the weight (as measured at the moment of the capture) and sex of the 23 G﻿PS﻿-tagge﻿d adult Andean condors from this study. Sex differences in weight of the sampled individuals conform mutually exclusive groups. The difference between the lightest (10.0 kg) and heaviest (15.68 kg) individuals exceeded 50%.

In addition, we hypothesize that the daily routines of condors vary with sex and body size as well as among seasons of the year. The Andean condor represents a very singular case of sexual size dimorphism in which males are up to 50% larger than females, whereas inter-individual differences within each sex can reach up to 25% in weight (Fig. 1). These differences drive dominance hierarchies in which larger males dominate smaller females in accessing resources^14^, but also different degree of dependence on wind resources as the sinking rate that a bird experiences increases with its body size^29^. Based on this, male condors would be expected to schedule their activities to maximize benefits from wind resources as they can access food wherever they arrive at a carcass. Within each sex, bigger birds should follow a similar strategy. Moreover, given that inbound flights (i.e. returning flights to nesting areas) require condors to undertake the energetically demanding task of climbing from low steppes to high mountains in the Andes, we expect that larger males schedule their routines especially to align these flights to the most profitable uplift conditions. On the other hand, we expect that condors adjust their routines to track seasonal changes in weather and daylight hours available to commute between nesting and foraging areas. By combining movement data with Bayesian hierarchical modeling, our study evaluates how different life-history constraints can act together to shape animal behavior and ecology.

## Results

**Figure 2:**
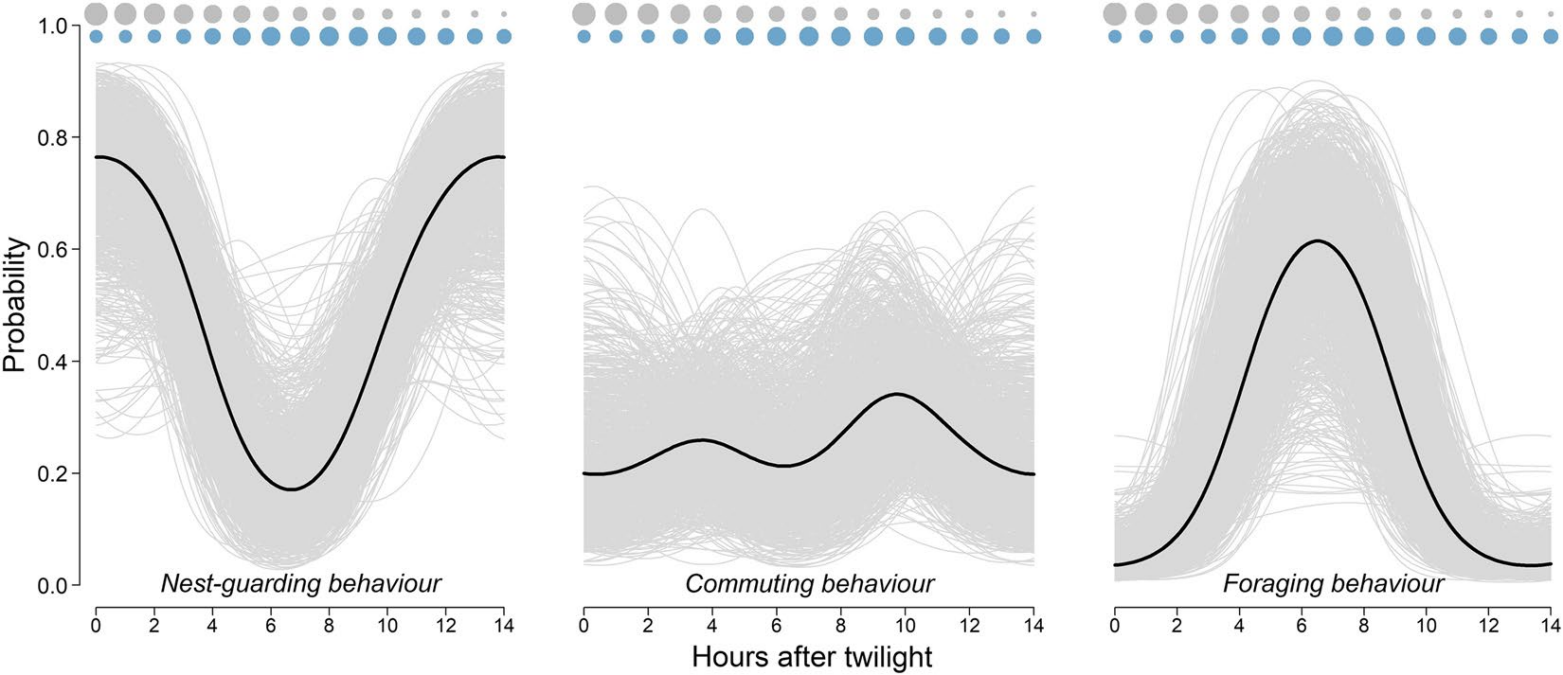
Daily routines of 17 GPS-tagged Andean condors considering the three most important behaviors: nest-guarding, commuting and foraging. Black lines represent mean values and light grey lines correspond to 1000 simulated curves from the posterior distributions of the estimated parameters. Superimposed on the routine are indicated the theoretical pattern of available carcass biomass over the day (grey circles) and the empirical pattern of wind speeds (blue circles) for the studied area. Note that the routine fits the idea of birds reaching an intermediate solution; i.e. searching for food in intermediate times between those optimal for finding food and use profitable wind resources.

Condors scheduled their routines in a regular way over the day (Fig. 2). As expected, the probability of nest-guarding and foraging showed a clear unimodal pattern whereas that of commuting varied in a less pronounced and bimodal fashion. During the first two daylight hours, the birds almost exclusively were in nest-guarding behavior from where they progressively switched to commuting. The probability of foraging peaked around six hours after twilight (i.e. around midday) and decreased thereafter. A second and slightly more pronounced peak in the probability of commuting occurred around ten hours after twilight corresponding to birds returning to nesting areas (Fig. 2).

**Figure 3:**
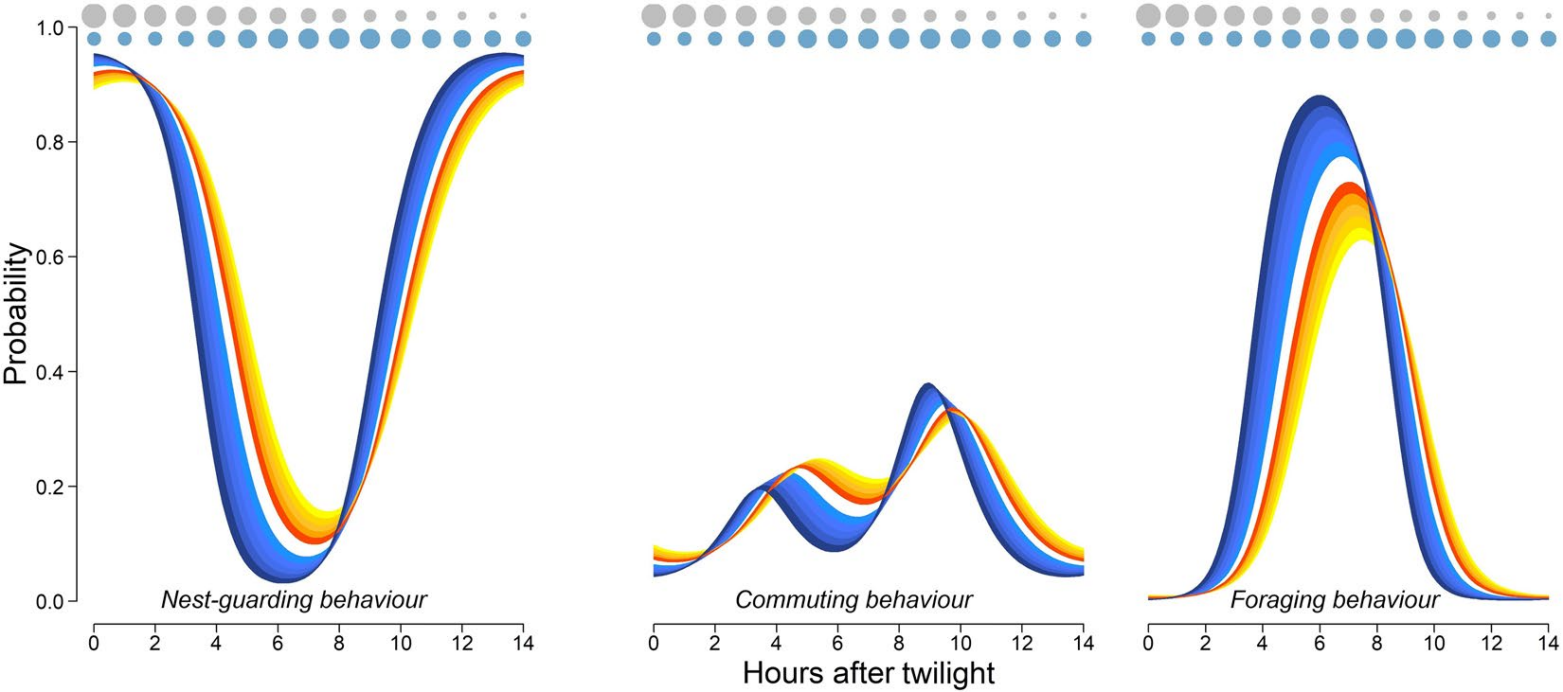
Influence of sex and body size (weight) on the daily routines of 17 GPS-tagged Andean condors. Blue gradient curves represent routines of males and red gradient curves represent those of females. The deeper the color, the heavier the individual is. Superimposed on the routine are indicated the theoretical pattern of available carcass biomass over the day (grey circles) and the empirical pattern of wind speeds (blue circles) for the studied area. Note that bigger birds show morning-skewed and less-time flexible routines in comparison to smaller birds. Also note that inbound flights of bigger birds are more closely align with the daily patterns of wind speed.

Sex and body size explained a large proportion of variability in daily routines (Fig. 3). In general, differences in sex and weight caused additive effects in condors’ routines, and no interaction between these variables was detected. Thus, routines of males tended to be much more skewed towards earlier hours in comparison to those of females, differing in almost two hours. Within each sex, heavier individuals showed more marked morning-skewed routines in comparison to lighter individuals. On the other hand, males showed less time-flexible routines compared to females, as evidenced by the more pronounced changes in their probability curves, similarly to that observed for the heavier individuals within each sex. All of these differences were evident for nest-guarding and commuting birds and were even more important for foraging birds (Fig. 3).

Daily routines were scheduled in such a way that foraging activities peaked at intermediate times of the day, w﻿hen the joint availability of carcas﻿ses and wind resources was the best for condors (Fig. 2). At the same time, this daily schedule led inbound flights to co-occur with the most profitable uplift conditions with those of larger individuals aligning more closely (Fig. 3).

**Figure 4:**
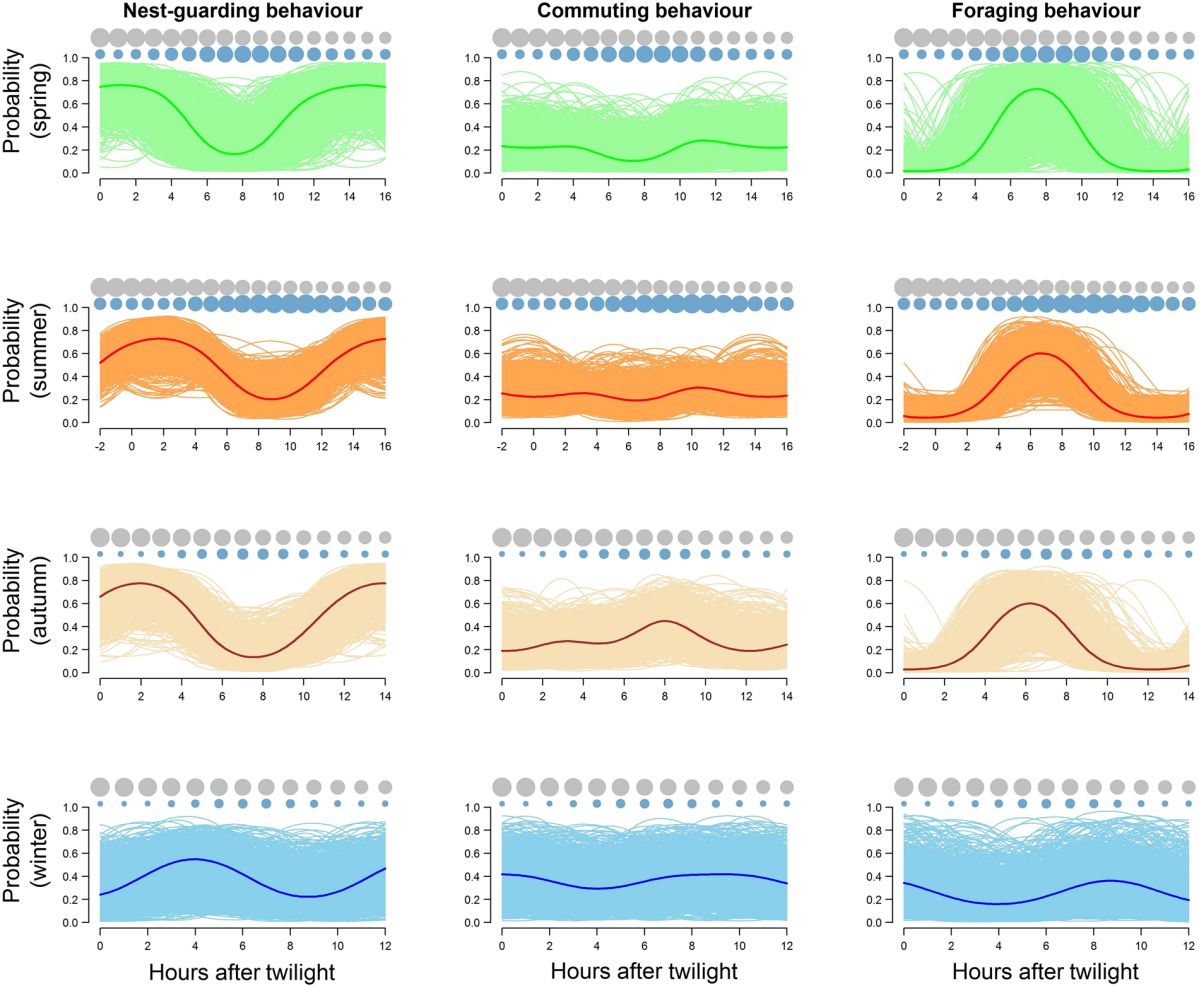
Seasonal variation in the daily routines of 17 GPS-tagged Andean condors considering the three most important behaviors: nest-guarding, commuting and foraging. Thicker lines represent mean values and thinner lines correspond to 1000 simulated curves from posterior distributions of estimated parameters. Superimposed on the routines are indicated the theoretical pattern of available carcass biomass over the day (grey circles) and the empirical patterns of wind speed (blue circle) for the studied area.

From summer onward, the length of daily routines contracted in accordance with the reduction in the available daylight hours (Fig. 4). Routines during spring, summer and autumn were similar to each other, but they differed from that during winter. Seasonal analysis also showed that inbound flights tended to be synchronized with the most profitable uplift conditions in each season (Fig. 4).

## Discussion

Andean condors scheduled their daily routines according to an intermediate solution strategy; i.e. they either did not venture out in search of food as early as the first sunlight was available or as late as the best uplift conditions occurred. Instead, they concentrated outbound flights, foraging and inbound flights on three, six and ten hours after twilight respectively, resulting in quite symmetric routines around the day. In addition, individuals of different sex and body size scheduled their activities differently, with routines becoming increasingly more morning-skewed and less-time flexible as body size increased. These differences resulted in a temporal partitioning between males and females due mainly to the pronounced sexual size dimorphism. Interestingly, these differences also led the birds to align inbound flights with the most profitable uplift conditions, particularly in the case of larger birds within each sex. Finally, daily routines were adjusted according to the variation in daylength and weather of each season of the year. As a whole, our results are consistent with the hypothesis that condors in Patagonia are subject to a trade-off between exploiting food and wind resources.

Previous studies on Old-World vultures support the idea that large avian scavengers are subject to a similar trade-off as their daily routines are also scheduled according to an intermediate solution strategy. For instance, griffon vultures (*Gyps fulvus*) typically start to forage around 2 hours after twilight (9.30 a.m.)^23^ whereas Cinereous vultures (*Aegypius monachus*) delay foraging between 2 and 3.5 hours after that moment (1.5 and 3 hours after sunrise), depending on the season^20^. White-backed vultures (*Gyps africanus*) and Ruppell’s vultures (*Gyps rueppelli*) in Namibia commonly leave the roost between 3 and 3.30 hours after twilight respectively^30^, whereas in Kenya, *Gyps* vultures are more abundant at carcasses 2 hours after that moment (8 a.m.)^24^. Most of these species feed on supplementary feeding stations that reduce the temporal unpredictability of food resources, conditions which have been found to reduce the urgency of birds to forage early in the morning^10, 31^. Andean condors may be experiencing similar foraging conditions as, in our study area, they exploit relatively abundant and predictable food sources that may allow them to forage later^32, authors unpub. data^. Delaying foraging activities may also be favored by the combination of two factors; the high inter-specific competitive ability of condors that allow them to displace other individuals from carcasses and the low densities of their populations that reduce the size of foraging groups^33, 34^.

Sexual size dimorphism often shapes a number of behavioral and ecological features of species^19, 35, 36^. The pronounced male-biased dimorphism in body size in the Andean condor underlies a social hierarchical system dominated by males that produce sex-specific differences in habitat use and accessing resources^14^. Our results suggest that, when combined with the trade-off between exploiting food and wind resources, sexual-size dimorphism in the Andean condor is also responsible for a partial temporal segregation between the sexes, which may be reducing agonistic encounters between them^37^. Moreover, the fact that females delay foraging activities may also stress the previously reported spatial partitioning^14^ as they would be forced to feed on carcasses avoided by males for being located in suboptimal places with higher levels of human-driven disturbance^32, 38^. As a result, this coexistence mechanism may come at high costs for females as they should schedule poorly efficient routines in energy terms and experiences higher risks at feeding sites. In fact, a recent study suggests that female Andean condors experience higher physiological costs than males, especially when the former need to take long flights to reach feeding grounds^39^.

The most energetically demanding movement phase for the studied birds is the return to their nesting areas. This is because condors have to climb from low- to high-altitude areas usually carrying full crops, conditions which importantly increases the sinking rate and ultimately the flight costs^40^. In line with this, our results show that condors take inbound flights concurrently with the most profitable uplift conditions probably because the use of wind resources offers them an energy-saving mechanism. In fact, the seasonal analysis suggests that the studied condors track the seasonal changes of uplift conditions to return their nests. The high energetic demand to return to nesting areas applies to every commuting condor but it should be even higher for the heavier ones^29^. To increase the chances of taking advantages of wind resources, it would then be expected that bigger birds are less flexible at the moment of taking inbound flights and this is precisely what our results show. Even when this strategy implies a suboptimal utilization of wind resources during other times of the daily schedule, it may be the one that offers the best net energy balance. For instance, when taking outbound flights, condors fly from their nesting areas which are in average located 320 m above the feeding area (1150 vs. 830 m a.s.l., data from this study) allowing them to use potential energy to subside, at least in part, the costs of transportation to the feeding areas.

As with many other species living in seasonal environments, the studied condors modified daily routines over the year to take advantages of all daylight hours available in each season^41, 42^. In winter, daily routines not only contracted but also showed an important variability that distorted the typical pattern found in the rest of the year. Distorted routines during winter could be caused by the urgency of obtaining food under almost permanently bad uplift conditions and shorter days, as suggested for other vulture species^20^.

An alternative explanation to the general pattern we found in condors’ daily routines could be that the birds are simply exploiting the whole time range with sunlight availability to symmetrically arrange activities around the day. Under this idea, however, it is more difficult to explain why the heavier birds showed markedly different routines compared to the smaller birds. As discussed above, this occurs even when heavier birds (large males) could delay foraging as they dominate intra-specific relationships^14, 33^. Therefore, we suggest that differences in the daily routines caused by inter-individual body size variation are revealing different degree of compromise when facing the trade-off between exploiting food and wind resources.

While behavioral trade-offs are a cornerstone in behavioral ecology and evolution, they are among the most challenging situations to empirically identify^6, 43^. Here we provide evidence supporting the existence of a trade-off in the Andean condor associated with the exploitation of key resources. This trade-off may have far-reaching implications for ecology and sociobiology of the species but also for its conservation. Trade-offs impose limitations on the capacity of organisms to respond to environmental changes^1, 44^. When variables involved in trade-offs are influenced by human activities the responsiveness may be even lower because changes occur more rapidly^45^. In the case of Andean condors, both food and wind resources are subject to change processes related to human activities^46, 47^ making them especially vulnerable to future scenarios as they might destabilize energy budgets and mechanisms of coexistence among individuals.

## Material and Methods

### Ethics Statement

This work was conducted in accordance with relevant national and international guidelines, and conforms to all legal requirements. Captures of the studied birds and all experimental procedures performed on them were duly approved by the Dirección de Fauna de Río Negro, Argentina (File number: 132730-DF-2010).

### Study area

This work was carried out in the Northwest Andean Patagonia of Argentina and Chile (38-42°S/69-72°W). The west side of this region is dominated by steep mountains and dense subantartic forests that abruptly change into flat and grassy steppes on the east side^48^. Extensive livestock production is one of the most important economic activities on the rangelands located on east of the region, with an average of 3.4 ha/LSU (LSU: livestock unit equivalent to one sheep weighting 40 kg)^49^. In this context, the steppes regularly provide condors with food resources whereas mountains in the west mainly serve as nesting and roosting sites.

The climate is temperate-cold with mean annual temperature values of about 8 °C (range 3–12 °C)^48^. A strong west-east gradient of precipitation crosses the region partly promoted by the predominance of winds from the west. Westerly winds constitute between 65 and 75% of daily observations in the year and their intensities range from 15 to 22 km/hr^48^. The area is characterized by marked seasonal patterns in temperature and rain with cold and rainy winters and hotter and drier summers^50^. The annual distribution of wind speed shows a maximum between spring and summer and a minimum in winter time. Wind speeds and temperatures follow hump-shaped patterns throughout the day, with low values during the early morning, increasing from that time to the afternoon and decreasing again in the evening (Supplementary Fig. S2).

### Study species

The Andean condor is the world’s heaviest soaring bird with individuals reaching up to almost 16 kg in weight (Fig. 1). This places them closest to the maximum size for flighted species. As a result, their movements are strongly influenced by the distribution of uplifts which is patchy both in space and time^26^. It is an obligate scavenger bird that mainly feeds upon carcasses of medium-to-large sized mammals. In the Andean Patagonia, the condors’ diet is mainly made up of domestic animals (i.e. sheep, *Ovis aries* and goat, *Capra hircus*) kept as livestock and secondarily of wild exotic species (i.e. hare, *Lepus europeus*, and red deer, *Cervus elaphus*)^46^. A large proportion of carcasses of these animals become available for scavenger species through the predation by pumas (*Puma concolor*) and foxes (*Lycalopex* spp.) which abandon them at their kills^51–53^. As these mammal predators show crepuscular and nocturnal hunting habits^51, 52^, carcass biomass available for diurnal scavenger species peaks during early morning hours and decays throughout the day.

### Data collection

Between October 2010 and January 2013, we trapped and tagged a total of 23 adult Andean condors with patagial PTT-100 50 g Solar Argos/GPS units from Microwave Telemetry Inc. (n = 10) and backpack 100 g Solar GPS–GSM CTT-1070-1100 units from CellTrack Inc. (n = 13) (see tagging details in ref. 54). These two types of units delivered one 3D GPS location every one hour and 15 minutes respectively, between the beginning and end of the astronomical twilight (i.e. times when the sun is 18 degrees below the horizon in the morning and evening, respectively).

### Data analysis

Most of the GPS-tagged Andean condors behave as central-place foragers making long commuting flights every two or three days between spatially well-defined nesting and foraging areas^54^. We used this spatial pattern to associate GPS locations with the three main behaviors in the daily routine of these birds: 1) ‘nest-guarding’, 2) ‘foraging’ and 3) ‘commuting’. The birds were assumed to be ‘nest-guarding’ anytime they were within their nesting areas either carrying out strictly reproductive activities (e.g. incubating) or others (e.g. resting). Nesting areas were defined as the 50% volume contours of Kernel Density Estimators^55^ and computed in ABODE^56^ using all available data for each bird. In the study area, condors primarily use the steppes to search for food and perch in large communal roosting sites^54, 57, 58^. For this reason, the birds were assumed to be ‘foraging’ anytime they visited the steppe except when they occasionally were within a 1 km radius of any communal roosting sites in this environment. Finally, we assumed that the birds were ‘commuting’ anytime they were between the areas above either taking outbound or inbound flights; i.e. flights from or to the nesting areas respectively. Since our main purpose was to evaluate how the birds allocate time to the different behaviors throughout the day, we only used movement data from the days when the individuals commuted between nesting and foraging areas. We conservatively excluded six individuals from statistical analyses as they showed no clear central-place foraging tactics, thus preventing us from reliably assigning GPS locations to the studied behaviors. After applying these criteria, our movement dataset contained a total of 8534 GPS locations from 17 individuals condors covering 250 days of the annual cycle.

To study how condors scheduled their daily routines, we first computed the hours passed after the morning civil twilight for every location (i.e. from now on ‘hours after twilight’) as from this moment on there is enough light to allow birds to fly. We then simultaneously modeled the probability that a bird is in anyone of the three behaviors (i.e. nest-guarding, foraging and commuting) as a sinusoidal function of the hours after twilight (*hours.after.twilight*). Thus, if *p*_[*t,k*]_ represents the probability that a bird is in behavior *k* as a function of time *t*, we can then express this function as follow:

1 $$logit({{\boldsymbol{p}}}_{[{\boldsymbol{t}},{\boldsymbol{k}}]})={{\boldsymbol{\beta }}}_{{\bf{0}}{\boldsymbol{k}}}+{{\boldsymbol{\beta }}}_{{\bf{1}}{\boldsymbol{k}}}\ast cos({{\boldsymbol{\beta }}}_{{\bf{2}}{\boldsymbol{k}}}+(\frac{\pi }{d{l}_{[t]}})\ast hours.after.twiligh{t}_{[t]})$$

In eq. (1) *β*_*0k*_ represents the baseline probability that a bird is in the behavior *k*, *β*_*1k*_ measures whether and how much such probability varies throughout the day and *β*_*2k*_ determines what time of day the probability reaches the maximum value. The time required to complete a cycle was scaled to the day length of every day in the year through the term *π/dl*_[*t*]_^59^. As condors could be in one of the different behaviors at a time *t*, we assumed that each observation was drawn from a multinomial distribution in which the *k* possible behaviors had probabilities *p*_*k*_ and where *p*_*1*_ + *p*_*2*_ … + *p*_*k*_ = 1^60^. Our model estimated the regression coefficients for *p*_*1*_ (probability of foraging) and *p*_*2*_ (probability of commuting) whilst *p*_3_ (probability of nest-guarding) was obtained as the residual probability 1 − *p*_*3*_ − *p*_*2*_. We wrote a system of equation to be sure that the sum of *p*_*k*_ reaches one. This model was constructed as a multilevel model with parameters varying at the individual level (see details in Supplementary Appendix I).

In order to evaluate how the general daily routines were affected by sex and body size, we expanded the model described above by incorporating the variables *sex* and *weight*, the latter containing the weight of the birds at the moment of capture (see details in Supplementary Appendix II). These variables were incorporated like linear predictors of the hyper-parameters of *β*_*1k*_ and *β*_*2k*_ (i.e. µ_*β1k*_ and µ_*β2k*_) as follow:

2 $${{\boldsymbol{\mu }}}_{{\boldsymbol{\beta }}{\bf{1}}{\boldsymbol{k}}[{\boldsymbol{j}}]}={{\boldsymbol{\omega }}}_{{\bf{0}}}+{{\boldsymbol{\omega }}}_{{\bf{1}}}\ast se{x}_{[j]}+{{\boldsymbol{\omega }}}_{2}\ast weigh{t}_{[j]}+{{\boldsymbol{\omega }}}_{{\bf{3}}}\ast se{x}_{[j]}\ast weigh{t}_{[j]}$$

3 $${{\boldsymbol{\mu }}}_{{\boldsymbol{\beta }}{\bf{2}}{\boldsymbol{k}}[{\boldsymbol{j}}]}={{\boldsymbol{\gamma }}}_{{\bf{0}}}+{{\boldsymbol{\gamma }}}_{{\bf{1}}}\ast se{x}_{[j]}+{{\boldsymbol{\gamma }}}_{{\bf{2}}}\ast weigh{t}_{[j]}+{{\boldsymbol{\gamma }}}_{{\bf{3}}}\ast se{x}_{[j]}\ast weigh{t}_{[j]}$$

Eq. (2) allowed us to test whether males and within males those heavier individuals showed less time-flexible routines, whilst eq. (3) tested whether routines of these birds were displaced in time compared to females and within females with those of lighter individuals. Note that both eqs (2) and (3) include interaction terms (ω_3_ and γ_3_) which allowed us to test if the effect of body size changed between sexes. To test the effect of seasonality, we modelled all the three parameters (*β*_*0k*_, *β*_*1k*_ and *β*_*2k*_) as linear functions of the observation-level variable *season* which contained the season of the year for each GPS location according to the Gregorian calendar. All the models were fitted using Bayesian statistics where priors for hyper-parameters were set as *t* distributions. We run three chains with 10000 iterations each and ruled out 5000 as burn-in. The models were implemented in JAGS via the ‘jagsUI’ package of R^61^.

After model fitting, we graphically compared the condors’ routines to the daily patterns of availability of food and wind resources. Given the lack of empirical data on the daily variation of carcasses in the study area, we assumed a linear decay model throughout the day. To describe the daily variation of wind resources, we used hourly wind speed data as a proxy of uplift availability. Specifically, we assumed that when wind speeds increased, so did the overall availability of uplifts. While this approach does not consider the fact that strong winds tend to break apart thermal formation^62^, it is still reasonable for our study as the condors mostly commute on an area dominated by a steep relief (Supplementary Fig. S3) that turns high-speed winds into strong orographic uplifts, thus compensating the possible degradation of thermals.

### Data availability

The datasets analysed during the current study are available from the corresponding author on reasonable request.

## Electronic supplementary material

Supplementary Information
