# Supplementary material for: Sexual-size dimorphism modulates the trade-off between exploiting food and wind resources in a large avian scavenger
Source: Sci Rep. 2017 Sep 13;7:11461. doi: 10.1038/s41598-017-11855-0 (PMC5597617; doi:10.1038/s41598-017-11855-0)
Supplement: Supplementary file 1 — Supplementary Information [file 41598_2017_11855_MOESM1_ESM.doc]

**Supplementary information**

**Sexual-size dimorphism modulates the trade-off between exploiting food and wind resources in a large avian scavenger**

Pablo A. E. Alarcón^*1,2,3^, Juan M. Morales^2^, José A. Donázar^4^, José A. Sánchez-Zapata^5^, Fernando Hiraldo^4^ & Sergio A. Lambertucci^1^

^(1)^ Grupo de Investigaciones en Biología de la Conservación, INIBIOMA (Universidad Nacional del Comahue-CONICET), Quintral 1250 (R8400FRF), Bariloche, Argentina.

^(2)^ Grupo de Ecología Cuantitativa, INIBIOMA (Universidad Nacional del Comahue-CONICET), Quintral 1250 (R8400FRF), Bariloche, Argentina.

^(3)^ The Peregrine Fund. 668 West Flying Hawk Lane, Boise, ID 83709, USA.

^(4)^ Department of Conservation Biology, Estación Biológica de Doñana, CSIC, E-41092 Sevilla, Spain.

^(5)^ Department of Applied Biology, University Miguel Hernández, E-03202 Alicante, Spain.

^*^Contact: [pabloalarcon@comahue-conicet.gob.ar](mailto:pabloalarcon@comahue-conicet.gob.ar)

**Figure S1.** Schematic representation of expected results under the three possible scenarios derived from the trade-off hypothesis. If searching for food resources is the priority for condors (‘food-priorization scenario’) the probability of being in the foraging behavior should fit closely to the availability pattern of food, while if saving energy subsiding the flight with wind uplifts is the priority (‘wind-priorization scenario’) the probability of being in the foraging behavior should fit more closely to the availability pattern of wind resource. A performance trade-off between these conflicting behavioral strategies would lead individuals to exploit sub-optimally both resources (‘intermediate-solution scenario’). x-axes encompass 18 hours of sunlight, period of time in which the birds are active.


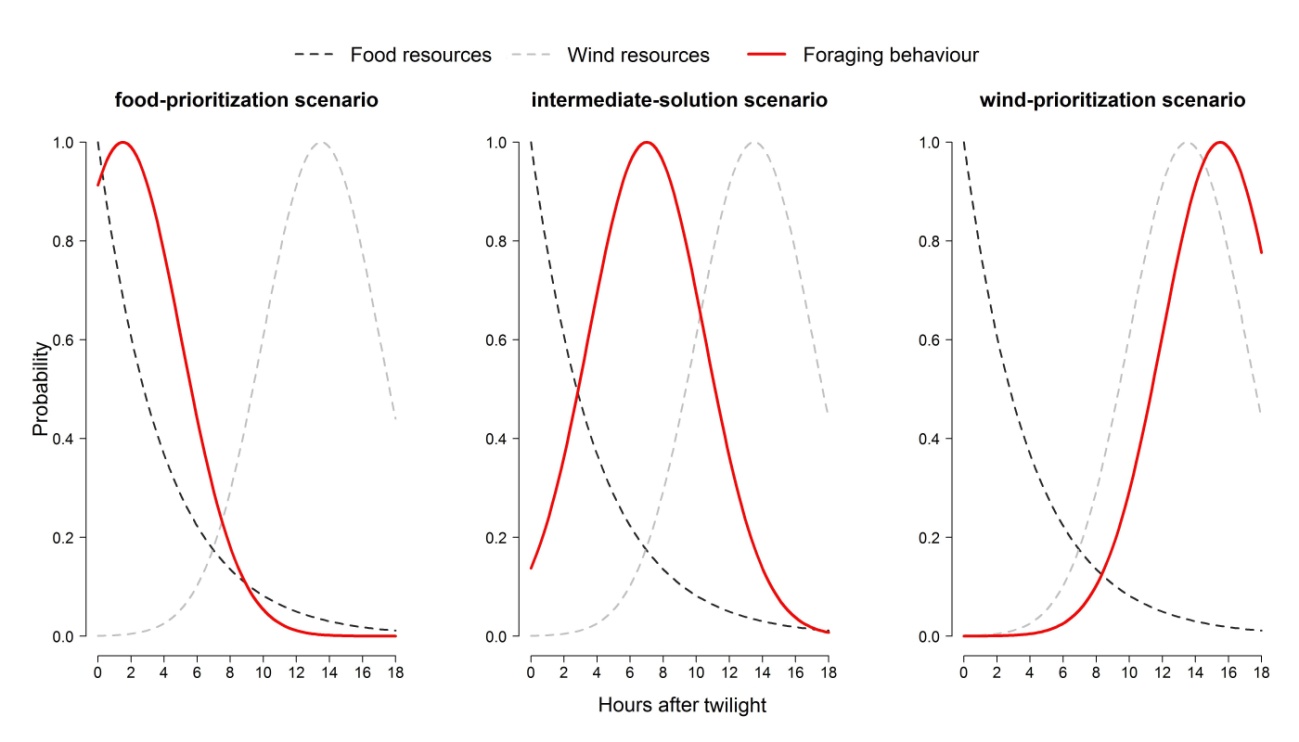


**Figure S2.** Daily pattern (mean ± sd) of wind speeds and temperatures in the study area. Data were recorded between January 2010 and December 2014 every 30 min at a meteorological station located in Bariloche City (41.139005 S, 71.327514 W).


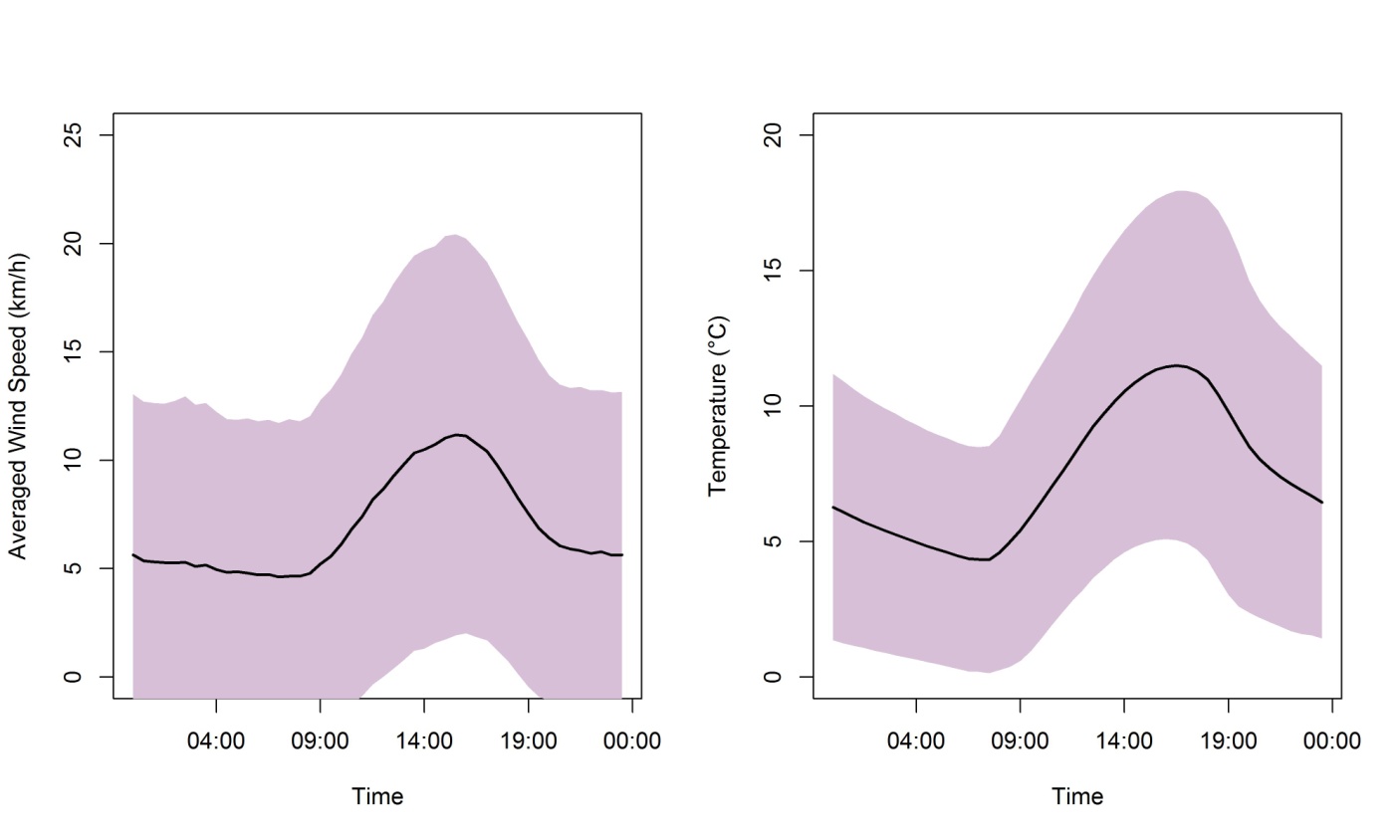


**Figure S3.** Movement dataset used to estimate daily routines of Andean condors in Patagonia. The entire dataset describing **nest-guarding, commuting and foraging behaviors covers very steep, densely forested mountains on the west side and flat, grassy steppes in the east side** (A)**. Three complete commuting flights from three different tracked individuals (B, C, D) show that they mainly breed and commute on mountain areas, whereas they use steppes to forage. Maps were created in R 3.1.1** ([http://www.R-project.org](http://www.R-project.org/)) **in combination with ArcGIS 10.1 (**[http://www.esri.com](http://www.esri.com/))**.**


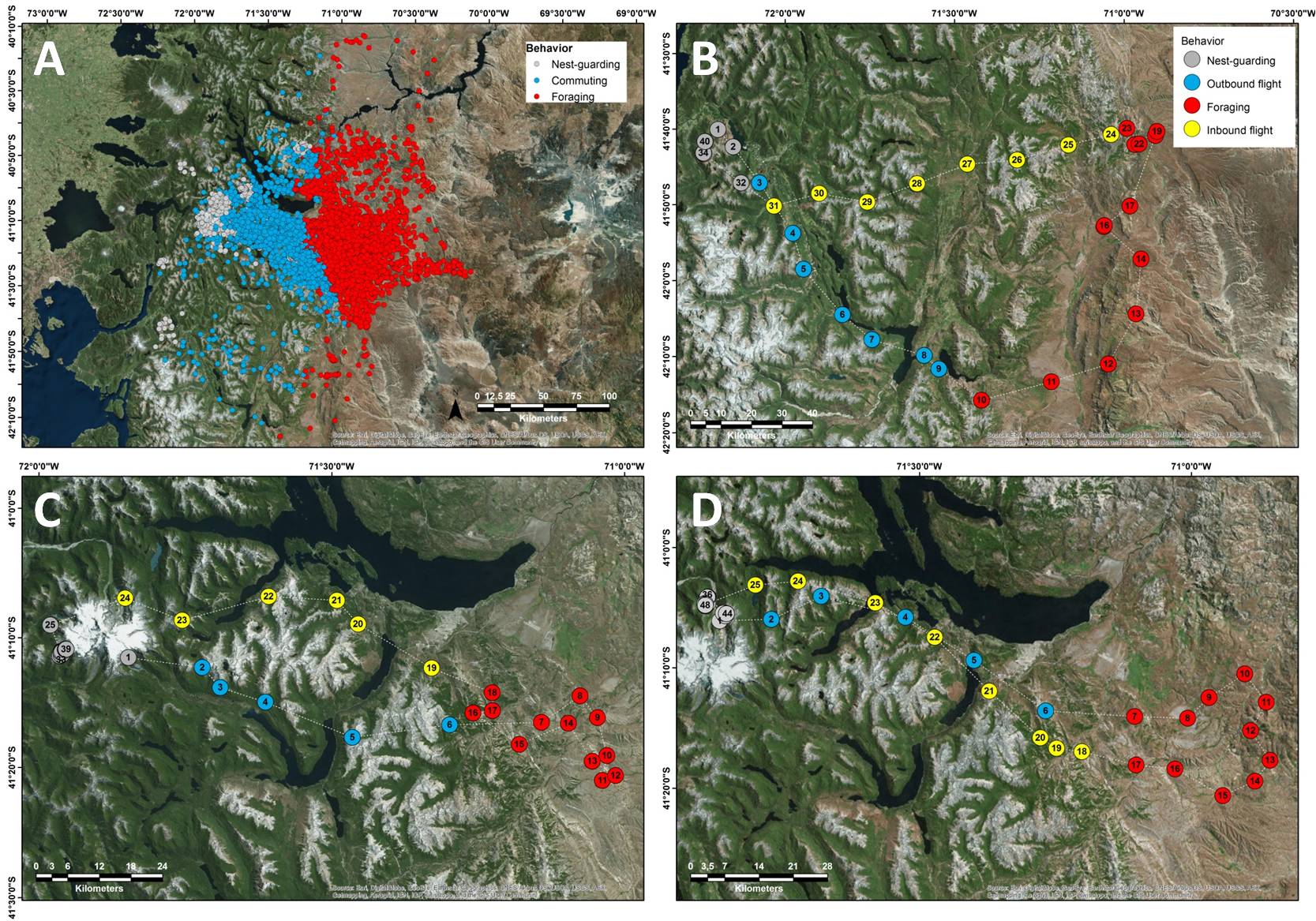


**Supplementary Appendix I.** BUGS code used to estimate the first model representing the daily routines of Andean condors. The same code was used to fit models to location data pooled by season.

model {

for(i in 1:ntot) {

#LIKELIHOOD FUNCTION

bahavior [i,1:3] ~ dmulti(p[i,1:3],nlocs[i])

logit(p[i,3]) <- b0_1[id[i]] + b1_1[id[i]] * cos(b2_1[id[i]] + (Pi/dl[i])* hrs.after.twilight[i])

p[i,2]<- (1 - p[i,3]) * q[i]

logit(q[i])<- b0_2[id[i]] + b1_2[id[i]] * cos(b2_2[id[i]] + (Pi/dl[i]) * hrs.after.twilight[i])

p[i,1] <- 1 - (p[i,2] + p[i,3])

}

#PRIORS AT THE INDIVIDUAL-LEVEL

for(k in 1:nid) {

b0_1[k] ~ dnorm(mu.b0_1, tau.b0_1)

b1_1[k] ~ dnorm(mu.b1_1, tau.b1_1)

b2_1[k] ~ dnorm(mu.b2_1, tau.b2_1) T(0,6.28)

b0_2[k] ~ dnorm(mu.b0_2, tau.b0_2)

b1_2[k] ~ dnorm(mu.b1_2, tau.b1_2)

b2_2[k] ~ dnorm(mu.b2_2, tau.b2_2) T(0,6.28)

}

# PRIOS AT THE GROUP-LEVEL

mu.b0_1 ~ dt(0,1,5)

sigma.b0_1 ~ dunif(1,5)

tau.b0_1<- 1/(sigma.b0_1*sigma.b0_1)

mu.b1_1 ~ dt(0,1,5)

sigma.b1_1 ~ dunif(1,5)

tau.b1_1<- 1/(sigma.b1_1*sigma.b1_1)

mu.b2_1 ~ dt(0,1,5)

sigma.b2_1 ~ dunif(1,5)

tau.b2_1<- 1/(sigma.b2_1*sigma.b2_1)

mu.b0_2 ~ dt(0,1,5)

sigma.b0_2 ~ dunif(1,5)

tau.b0_2<- 1/(sigma.b0_2*sigma.b0_2)

mu.b1_2 ~ dt(0,1,5)

sigma.b1_2 ~ dunif(1,5)

tau.b1_2 <- 1/(sigma.b1_2*sigma.b1_2)

mu.b2_2 ~ dt(0,1,5)

sigma.b2_2 ~ dunif(1,5)

tau.b2_2 <- 1/(sigma.b2_2*sigma.b2_2)

Pi<-3.141593

}

**Supplementary Appendix II.** BUGS code used to estimate the model including the body weight and sex effects on the daily routines of Andean condors.

model {

# LIKELIHOOD FUNCTION

for(i in 1:ntot) {

bahavior [i,1:3] ~ dmulti(p[i,1:3],nlocs[i])

logit(p[i,3]) <- b0_1[id[i]] + b1_1[id[i]] * cos(b2_1[id[i]] + (Pi/dl[i])* hrs.after.twilight[i])

p[i,2]<- (1 - p[i,3]) * q[i]

logit(q[i])<- b0_2[id[i]] + b1_2[id[i]] * cos(b2_2[id[i]] + (Pi/dl[i]) * hrs.after.twilight[i])

p[i,1] <- 1 - (p[i,2] + p[i,3])

}

# PRIORS AT THE INDIVIDUAL-LEVEL

for(k in 1:nid) {

b0_1[k] ~ dnorm(mu.b0_1, tau.b0_1)

b1_1[k] ~ dnorm(mu.b1_1[k], tau.b1_1)

b2_1[k] ~ dnorm(mu.b2_1[k], tau.b2_1) T(0,6.28)

b0_2[k] ~ dnorm(mu.b0_2, tau.b0_2)

b1_2[k] ~ dnorm(mu.b1_2[k], tau.b1_2)

b2_2[k] ~ dnorm(mu.b2_2[k], tau.b2_2) T(0,6.28)

mu.b1_1[k] <- om0_1 + om1_1 * sex[k] + om2_1 * weight[k] + om3_1 * sex[k] * weight[k]

mu.b2_1[k] <- gam0_1 + gam1_1 * sex[k] + gam2_1 * weight[k] + gam3_1 * sex[k] * weight[k]

mu.b1_2[k] <- om0_2 + om1_2 * sex[k] + om2_2 * weight[k] + om3_2 * sex[k] * weight[k]

mu.b2_2[k] <- gam0_2 + gam1_2 * sex[k] + gam2_2 * weight[k] + gam3_2 * sex[k] * weight[k]

}

# PRIORS AT GROUP LEVEL

mu.b0_1 ~ dt(0,1,5)

sigma.b0_1 ~ dunif(1,5)

tau.b0_1 <- 1/(sigma.b0_1*sigma.b0_1)

mu.b0_2 ~ dt(0,1,5)

sigma.b0_2 ~ dunif(1,5)

tau.b0_2 <- 1/(sigma.b0_2*sigma.b0_2)

om0_1 ~ dt(0,1,5)

om1_1 ~ dt(0,1,5)

om2_1 ~ dt(0,1,5)

om3_1 ~ dt(0,1,5)

gam0_1 ~ dt(0,1,5)

gam1_1 ~ dt(0,1,5)

gam2_1 ~ dt(0,1,5)

gam3_1 ~ dt(0,1,5)

om0_2 ~ dt(0,1,5)

om1_2 ~ dt(0,1,5)

om2_2 ~ dt(0,1,5)

om3_2 ~ dt(0,1,5)

gam0_2 ~ dt(0,1,5)

gam1_2 ~ dt(0,1,5)

gam2_2 ~ dt(0,1,5)

gam3_2 ~ dt(0,1,5)

Pi<-3.141593

}
